# Transdermal microneedle integrating a biomimetic self-enhancing Fenton reaction nano-reactor for alleviating rheumatoid arthritis by inflammatory microenvironment remodeling

**DOI:** 10.7150/thno.114855

**Published:** 2025-06-18

**Authors:** Ying Gao, Xuejun Chen, Lin Liu, Jingya Xiu, Yufei Wen, Chunrong Yang, Degong Yang, Fen Yao

**Affiliations:** 1 Department of Pharmacy, Shantou University Medical College, Shantou, 515041, China.; 2 Department of Pharmacy, Department of Dermatology, The First Affiliated Hospital of Shantou University Medical College, Shantou, 515041, China.; 3 Department of Pharmacy, The Second Affiliated Hospital of Shantou University Medical College, Shantou, 515041, China.

**Keywords:** inflammation, ROS scavenging, M2 macrophage polarization, reducing hypoxia

## Abstract

**Rationale:** Rheumatoid arthritis (RA) is a chronic autoimmune inflammatory disease, and persistent inflammation in multiple joints is an important sign for the progression of RA. To this end, we developed the transdermal microneedle integrating biomimetic self-enhancing Fenton reaction nano-reactor, for the purposes of eliminating reactive oxygen species, reducing hypoxia and inflammation, and regulating macrophage phenotype.

**Methods:** A novel biomimetic self-enhanced Fenton reaction nano-reactor was synthesized using an M1 macrophage cell membrane-coated tannic acid-modified iron oxide nanoparticle (IO-NH_2_-TA TNPs@M1). The regulatory mechanisms of the IO-NH_2_-TA TNPs@M1 were investigated by evaluating ROS scavenging, degree of hypoxia, adsorption of pro-inflammatory factors, and M2 macrophage polarization. Then, the nano-reactor was incorporated into a dissolving microneedle, utilizing enzyme-cut oligomeric sodium hyaluronate, and subsequently assessed for pharmacodynamics and safety.

**Results:** *In vitro* mechanisms of IO-NH_2_-TA TNPs@M1 included eliminating ROS, inhibiting the expression of HIF-1α, decreasing the content of pro-inflammatory factors (IL-6 and TNF-α), and inducing macrophage M2 polarization. Pharmacodynamic and *in vitro* mechanistic studies showed that IO-NH_2_-TA TNPs@M1DM maximally alleviated joint swelling and fever, protected joint cartilage, improved the local hypoxia environment and promoted macrophage M2 polarization. Cytotoxicity assays and HE staining showed that IO-NH_2_-TA TNPs@M1DM displayed good biocompatibility.

**Conclusions:** This study designed and synthesized an innovative biomimetic self-enhancing Fenton reaction nano-reactor, and utilized microneedles for the transdermal delivery, providing a scientific and effective new strategy for the precise treatment of RA.

## Introduction

Rheumatoid arthritis (RA) is a chronic immune-mediated systemic inflammatory disease that typically results in localized damage of articular cartilage, bone and tendons 1. Chronic synovitis and cell infiltration are prominent pathological features associated with RA 2. According to statistics, approximately 1% of the global population is affected by RA. On average, the life span of patients is shortened by 5-10 years, and the standard incidence of mortality exceeds 1.5%, which has emerged as a significant contributor to human disability and loss of productivity 3.

Though many biomolecular mechanisms have been proposed, the pathogenesis of RA is complex and its etiology has not been fully elucidated. When RA occurs, CD4^+^T cells play an important role in stimulating synovial macrophages and fibroblast-like synovial cells by stimulating the expression of nuclear factor kB and the production of interleukin 17, thereby promoting inflammation, bone erosion and cartilage degradation 4. Moreover, reactive oxygen species (ROS) and proteases are produced by neutrophils in synovial fluid, which cause oxidative stress and local hypoxia, leading to bone erosion and cartilage degradation 5. In summary, the primary pathological features of RA are known, and include sustained inflammation in joint cavities, excess ROS and local hypoxia 6.

At present, drugs used for RA mainly relieve inflammation and improve joint mobility, and are divided into three categories: disease-modifying antirheumatic drugs (DMARDs), non-steroidal anti-inflammatory drugs (NSAIDs) and glucocorticoids (GCs) 7. However, current drug treatment is accompanied by serious adverse effects, including gastrointestinal complications, hepatotoxicity, tuberculosis, and shingles infections 8. Due to the emergence of drug resistance and the transient nature of remission, patients are subjected to the discomfort associated with the administration of high doses and frequent usage of pharmacological agents. Furthermore, achieving a holistic regulation of the various pathogenic mechanisms of RA presents significant challenges 9. On the other hand, it is challenging for drug to selectively target the lesion site, resulting in the above adverse reactions 10. The existing practice of administering treatments directly into the articular cavity primarily serves to provide temporary local relief of symptoms. Consequently, the advancement of effective and safe treatments for RA holds significant research importance and clinical relevance.

Nano-based drug delivery systems have attracted wide attention in the field of medical research, due to their advantages in targeted delivery, response to drug release, and improvement of drug solubility 11, and have been successively applied in RA treatment. Polymer-modified DNA hydrogels have been designed, in which a nanozyme is used to clear excess ROS from the RA microenvironment and reduce inflammation, and intracellular transmission of living cell mitochondria is applied to inhibit ROS production 12. Another study eliminated the inflammatory response in RA by promoting M2 macrophage polarization, where novel core-shell nanocomplexes were designed and synthesized using a quadrilateral-shaped ruthenium nanoparticle (core), and a poly (lactate-glycolic acid) modified by molecular glucan sulfate (shell). These nanocomplexes markedly enhanced the targeting efficiency of resveratrol and polarization of M2 macrophage, resulting in precise localization at the lesion sites, and mitigation of the inflammatory response associated with RA 13. While the established nano-based drug delivery systems improve the therapeutic effect of RA, challenges remain in concurrently ameliorating the pathological characteristics of RA-affected tissue. Consequently, this study seeks to regulate the microenvironment of the RA lesion, from the perspective of "multiple targets and multiple pathways", to improve therapeutic outcomes of pharmacological interventions in RA.

Dissolving microneedles (DMs) have garnered much attention due to their ideal transdermal delivery efficiency and minimal adverse effects 14. DMs are composed of biocompatible and water-soluble material, and dissolve in the skin interstitial fluid for releasing drugs at the target site, and are gradually being applied in the treatment of RA 15. A programmable polymer DMs-platform has been developed for transdermal delivery of methotrexate and a ROS scavenger (polydopamine/manganese dioxide), effectively clearing ROS and reducing RA inflammation, thus enhancing RA therapy 16. Especially for biologics with high molecular weight, low drug transdermal delivery efficiency results from the relatively strong skin barrier. To this end, hyaluronic acid microneedles containing melittin have been prepared that for RA treatment, which reduce levels of pro-inflammatory cytokines (IL-17 and TNF-α) and increased the percentage of CD4^+^T cells 17. Therefore, DMs made of water-soluble excipients have the advantages of injection and transdermal drug delivery, effectively addressing the challenges associated with transdermal administration of nanodrugs.

Iron oxide nanoparticles (IO NPs) have no toxic side effects and have a surface easy to modify for binding to a variety of substances, such as proteins, polysaccharides, and polyethylene glycol 18. Crucially, iron ion is released after IO NPs enter the weakly acidic microenvironment, which catalyzes the Fenton reaction to produce oxygen at the lesion site, thus consuming ROS and alleviating hypoxia 19,20. However, IO NPs face limitations in their application for drug delivery due to a deficiency in active sites for drug loading 21. So, silane coupling agents were used to modify IO NPs through an alkylation reaction (IO-NH_2_ NPs), which increased the number of amino groups and provided support for their application in drug delivery 22. To further enhance the Fenton reaction of IO-NH_2_ NPs, tannic acid (TA) as the reducing agent was used to accelerate the conversion of Fe^3+^/Fe^2+^, thereby achieving sustainable and efficient Fenton-mediated chemokinetic therapy (IO-NH_2_-TA NPs) 23.

The inhibition of intracellular signaling pathways associated with inflammatory cytokines has been demonstrated to be an effective approach for the treatment of RA 24. Tofacitinib (Tof), a JAK1 inhibitor, is used to treat the RA. JAK kinases play an important role in the signaling pathways of various cytokines, growth factors and hormones, and are closely related to the pathogenesis of autoimmune inflammatory diseases. Selective inhibition of JAK1 blocks signaling pathways associated with pathogenic cytokines in autoimmune inflammatory disease, while preserving other signaling pathways required for normal physiological function 25. Therefore, we chose Tof as a model drug due to its rapid onset of action and distinctive mechanism of action (IO-NH_2_-TA TNPs).

Moreover, during the development of RA, synovial tissue macrophages are the crucial immune cells responsible for mediating joint inflammation. Macrophages are divided into pro-inflammatory M1-type macrophages and anti-inflammatory M2-type macrophages 26. Therefore, IO NPs can also be used to modulate the RA microenvironment by inducing the conversion of M1 to M2 macrophages for the treatment of RA 27. Thus, M1 macrophage membranes were used to encapsulate IO-NH_2_-TA, creating a biomimetic self-enhancing Fenton reaction nano-reactor (IO-NH_2_-TA TNPs@M1), to adsorb various pro-inflammatory factors at the lesion site and remodel the inflammatory microenvironment of RA.

In order to achieve precise transdermal delivery in the joint cavity, IO-NH_2_-TA TNPs@M1s, were incorporated into DMs (to form IO-NH_2_-TA TNPs@M1DMs) prepared by enzyme-cut oligomeric sodium hyaluronate, where sodium hyaluronate, a natural high molecular weight polysaccharide, was degraded into oligo-sodium hyaluronate by enzyme digestion, making it more easily absorbed by the skin 28. After injection into the skin, the oligomeric sodium hyaluronate will gradually dissolve under the action of skin tissue fluid, and release the IO-NH_2_-TA TNPs@M1 into the skin for transdermal delivery.

**Figure 1:**
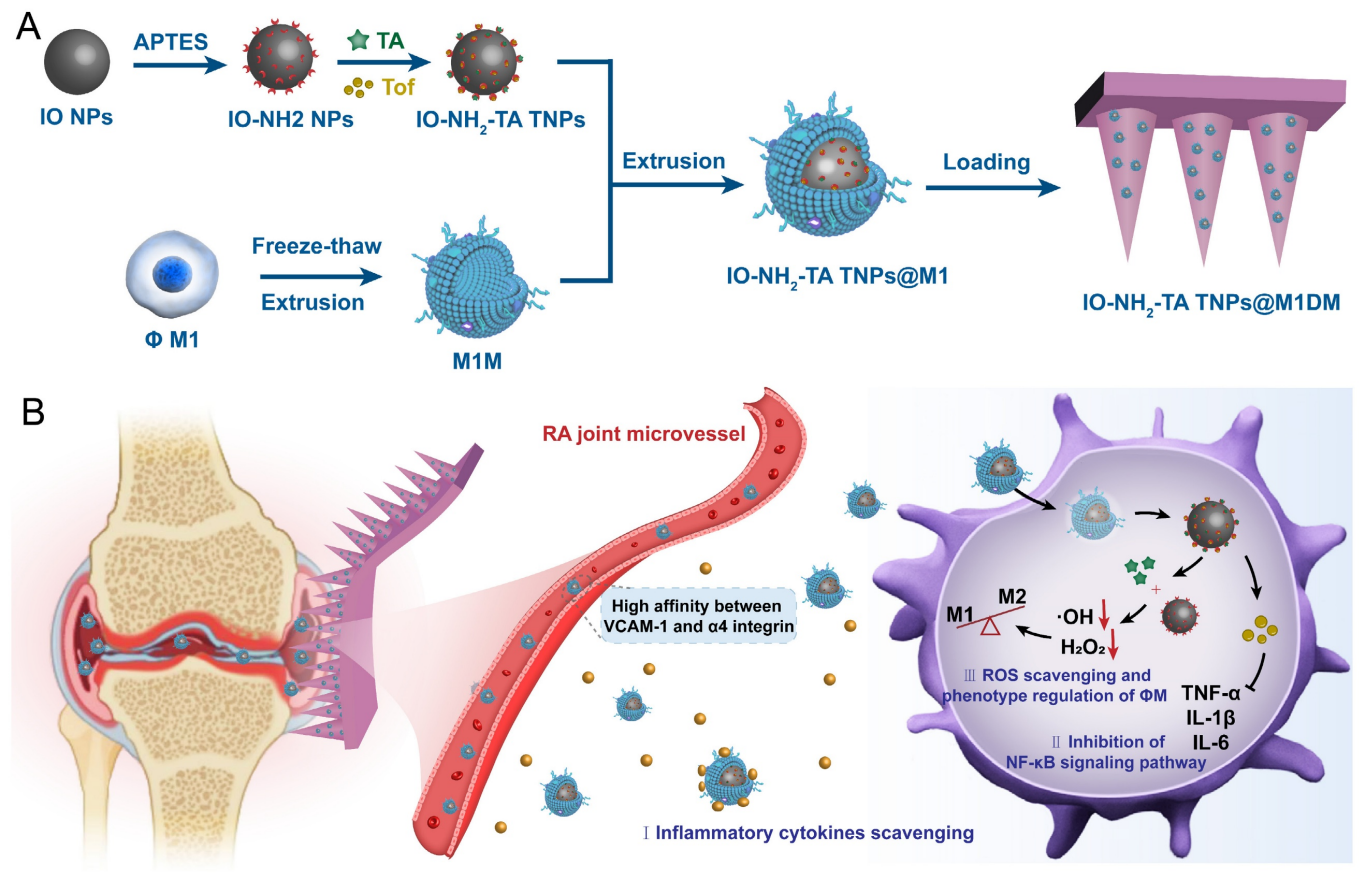
Schematic illustration of preparation and mechanisms of IO-NH_2_-TA TNPs@M1DMs.

Therefore, this study designs and synthesizes a new biomimetic self-enhancing Fenton reaction nano-reactor by using a nano-drug delivery system, suited for the pathological characteristics of RA (Figure 1), and employs a DM for accurate transdermal administration in the management of RA. The drug delivery system aims to achieve the purposes of blocking the inflammatory response, regulating macrophage phenotype, clearing reactive oxygen species and improving local anaerobic environment, which will provide a new scientific and effective strategy for the precise treatment of RA.

## Results and Discussion

### Characterization of IO-NH_2_-TA TNPs

**Figure 2:**
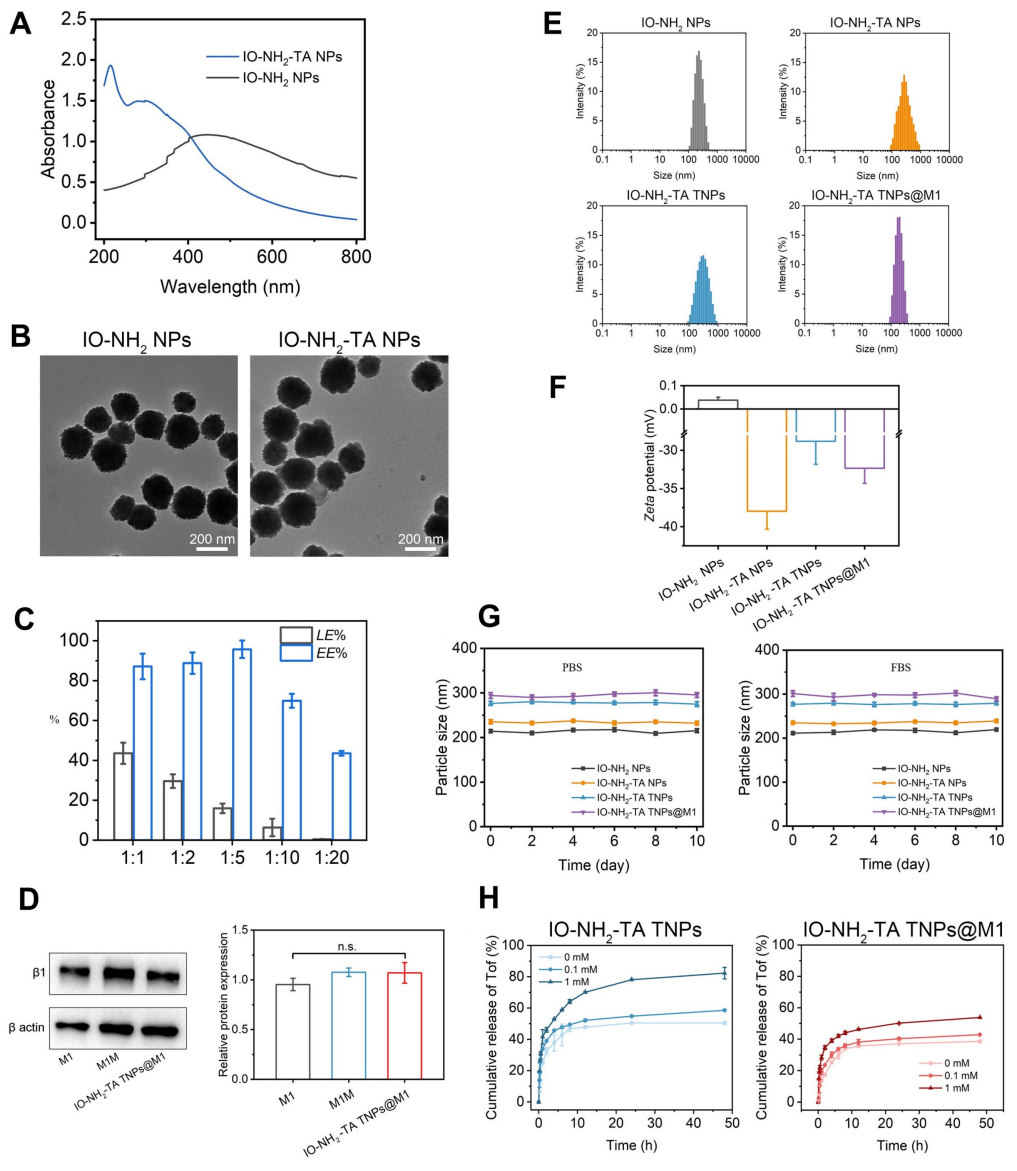
UV spectra of IO-NH_2_ NPs and IO-NH_2_-TA NPs (A). TEM images of IO-NH_2_ NPs and IO-NH_2_-TA NPs (B). Drug loading and encapsulation efficiency values of NPs formed at diverse ratios (w/w) of IO-NH_2_-TA TNPs (C). SDS-PAGE protein profile of M1, M1M, and IO-NH_2_-TA TNPs@M1 (D). Size distribution, zeta potential and stability of IO-NH_2_ NP, IO-NH_2_-TA NPs, IO-NH_2_-TA TNPs and IO-NH_2_-TA TNPs@M1 (E-G). Drug release of IO-NH_2_-TA TNPs and IO-NH_2_-TA TNPs@M1 at different concentrations of H_2_O_2_ (H).

Full-wavelength scanning of the IO-NH_2_ NPs and IO-NH_2_-TA NPs was conducted by UV spectrophotometry (Figure 2A). IO-NH_2_ NPs had an absorption peak at 450 nm. However, corresponding absorption peaks at 215 nm and 298 nm appeared for IO-NH_2_-TA NPs, and the absorption peak at 450 nm disappeared, indicating that TA was successfully incorporated into the IO-NH_2_ NPs. TEM images of IO-NH_2_ NPs and IO-NH_2_-TA NPs were showed in Figure 2B, and appeared as solid-core spherical or sphere-like structures. In addition, the surface of IO-NH_2_-TA NPs existed as a light-colored film, indicating that IO-NH_2_-TA NPs were successfully prepared. Raman spectroscopy was further used to verify the successful synthesis of IO-NH_2_-TA NPs (Figure S1), and showed peaks at 1451 and 1357 cm^-1^, which corresponded to the C-C bond of benzene ring and the phenolic hydroxyl group, respectively, characteristic of TA 29. These two peaks also appeared in the Raman spectra of IO-NH_2_-TA TNPs (1470 and 1349 cm^-1^), confirming that the IO-NH_2_ NPs were modified by TA. Using a similar method, TA-modified Fe_3_O_4_ nanoparticles loaded with gold were also prepared, the stable shell layer was formed by coordination of the polyphenolic groups of TA with Fe^3+^ 30.

Firstly, Raman spectroscopy was used to verify the successful drug loading of IO-NH_2_-TA TNPs. As shown in Figure S1, the characteristic peaks of Tof were observed at 1233 and 1570 cm^-1^, which correspond to the pyrrole ring and the C-C ring, respectively 31. The above peaks were located at 1243 and 1576 cm^-1^ in the Raman spectrum of IO-NH_2_-TA TNPs, indicating that Tof was successfully loaded onto the nanoparticles. Then, the drug loading and encapsulation rate of IO-NH_2_-TA TNPs were determined (Figure 2C). With the increase of the proportion of drug mass, drug loading gradually increased, but the encapsulation rate gradually decreased due to the limited binding sites. Therefore, an optimal mass ratio of drug to carrier ratio of 1:5 was selected. In this study, drug loading of IO-NH_2_-TA TNPs was increased through modification by introducing an active functional group (-NH_2_). It had been previously shown that amino-modified Fe_3_O_4_ nanoparticles were compounded with a cationic polymer for delivering DNA/siRNA to increase drug loading to 40%, remarkedly higher than that of traditional cationic polymers owing to increasing active functional groups 32.

### Characterization of IO-NH_2_-TA TNPs@M1 nanoparticles

Western blotting was used to analyze membrane protein expression, including M1 cell lysate, M1 cell membrane, and IO-NH_2_-TA TNPs@M1 nanoparticles. As shown in Figure 2D, M1 membrane proteins expressed in the M1 group, M1M group, and IO-NH_2_-TA TNPs@M1 had no significant difference (*p* > 0.05), indicating that the M1M protein of nanoparticles was structurally intact and functionally normal 33.

Particle size distribution and zeta potential of the nanoparticles (IO-NH_2_ NPs, IO-NH_2_-TA NPs, IO-NH_2_-TA TNPs and IO-NH_2_-TA TNPs@M1) were characterized using a Malvern particle size analyzer. Particle sizes of the above nanoparticles were uniform and homogeneous, and the *PDI* was less than 0.2 (Figure 2E, Table S1). Particle size increased with TA modification, Tof loading and M1 cell membrane coating. The zeta potentials of the nanoparticles were shown in Figure 2F and Table S1. The zeta potential of IO-NH_2_ NPs was 0.00 ± 0.12 mV owing to the amino group on the surface. The zeta potential of IO-NH_2_-TA NPs was -37.97 ± 2.34 mV, due to the introduction of TA 34. The Zeta potential of IO-NH_2_-TA TNPs was -28.81 ± 3.01 mV, and that of IO-NH_2_-TA TNPs@M1 was -32.34 ± 1.97 mV, which could be attributed to the protein of the biofilm.

The PBS was consistent with the physiological environment and suitable for simulating the *in vivo* environment. The serum protein in FBS was rapidly adsorbed to the surface of nanoparticles to form a "protein crown" that inhibited aggregation. IO-NH_2_ NPs, IO-NH_2_-TA NPs, IO-NH_2_-TA TNPs and IO-NH_2_-TA TNPs@M1 nanoparticles, in PBS and FBS, displayed good stability because the particle sizes were not changed (Figure 2G).

To approximate drug release from IO-NH_2_-TA TNPs in the oxidative RA microenvironment, we exposed IO-NH_2_-TA TNPs to different concentrations of H_2_O_2_ and measured Tof release from IO-NH_2_-TA TNPs (Figure 2H). Drug release increased with the increase of H_2_O_2_ concentration, which was significantly faster than in a normal physiological environment (*p* < 0.05). This was due to the Fenton reaction, which controlled drug release in RA join and avoided drug leakage in normal tissues. Drug release of IO-NH_2_-TA TNPs@M1 nanoparticles was decreased compared with IO-NH_2_-TA TNP, which was caused by the encapsulation of cell membrane and hindered the release of drugs (Figure 2H).

### *In vitro* mechanisms by which IO-NH_2_-TA TNPs@M1 regulate the RA microenvironment

**Figure 3:**
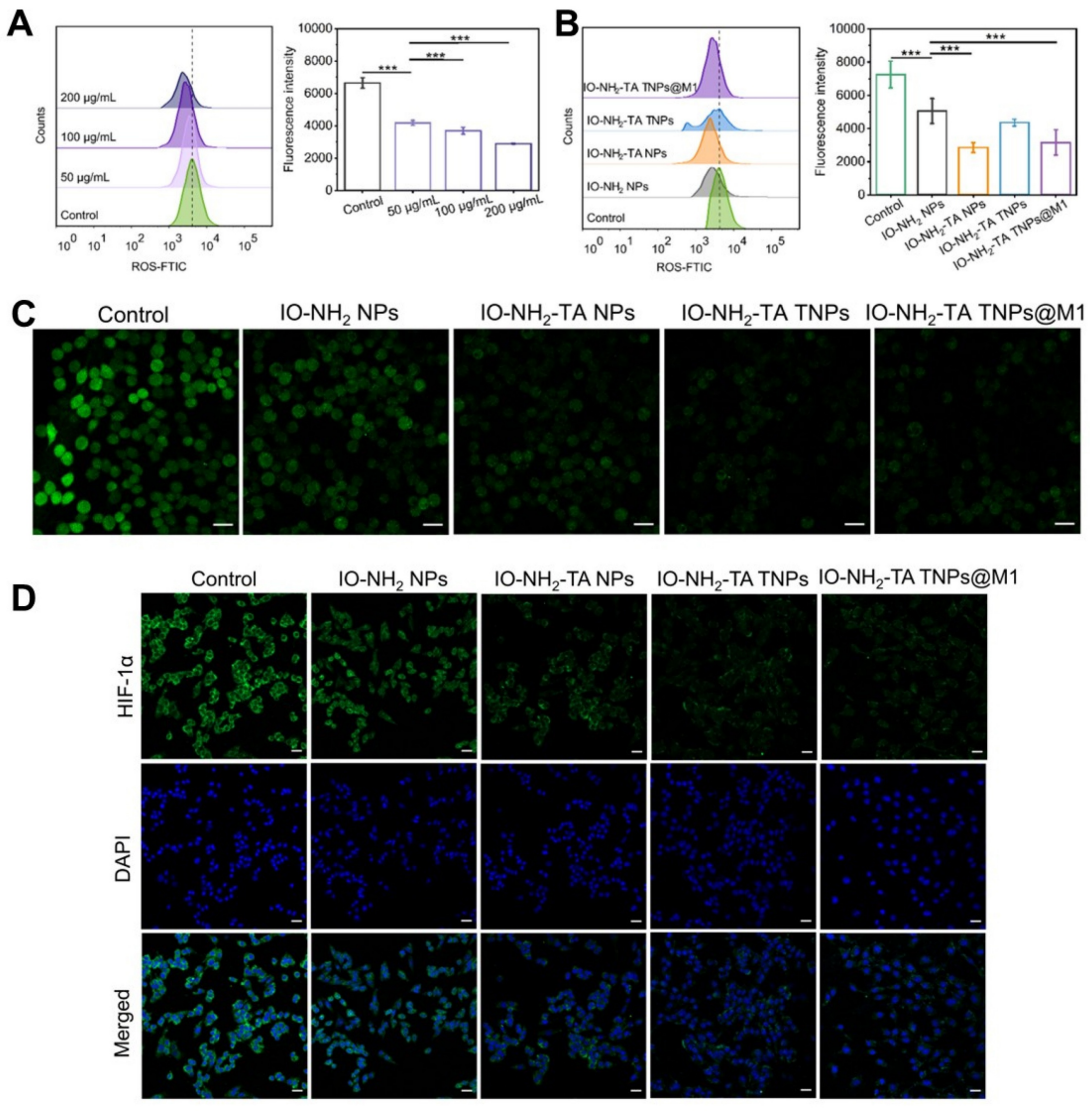
Flow cytometry analysis (A-B) and confocal laser scanning microscopy images (C) of ROS in MH7A cells under various treatments. Scale bars, 20 µm. Immunofluorescence staining of HIF-1α (D) in MH7A cells under various treatments. Scale bars, 20 µm.

ROS scavenging of IO-NH_2_-TA TNPs@M1 was evaluated using RA synovial fibroblasts (MH7A cells) 35. Compared with the control group, intracellular ROS as measured by flow cytometry was reduced after treatment with IO-NH_2_ NPs (*p* < 0.001), indicating that IO-NH_2_ TNPs had good ROS scavenging ability, and increased with the increase in concentration (Figure 3A). In addition, the ROS scavenging ability was enhanced after TA modification (*p* < 0.001), further suggesting that TA enhanced the Fenton reaction (Figure 3B) 23. Confocal laser scanning microscopy also showed that the fluorescence of control group was strongest, followed by the IO-NH_2_ NP group, and then the other groups modified with TA (Figure 3C). For investigating ferroptosis-inducing capability of IO-NH_2_-TA TNPs@M1 nanoparticles, we measured the levels of GPX4 secreted by MH7A cells co-cultured with the nanoparticles (Figure S2). Compared to the blank group, the GPX4 levels in cells treated with nanoparticles were reduced (*p* < 0.01). Above all, this suggested that IO-NH_2_-TA TNPs@M1 had the ROS scavenging and ferroptosis-inducing activity.

The local hypoxia environment of RA joint causes the up-regulation of HIF-1α expression, and HIF-1-induced pro-inflammatory factors, further activating immune cells, and resulting in an inflammatory cascade. It was also elucidated that leflunomide and HIF-1α inhibitors reduced bone erosion in RA with C-reactive protein abnormalities 36. Therefore, HIF-1α content plays a key role in understanding the pathogenesis of RA. Immunofluorescence demonstrated IO-NH_2_-TA TNPs@M1s clearly inhibited the expression of HIF-1α (Figure 3), suggesting that oxygen was generated and improved the local hypoxia environment.

**Figure 4:**
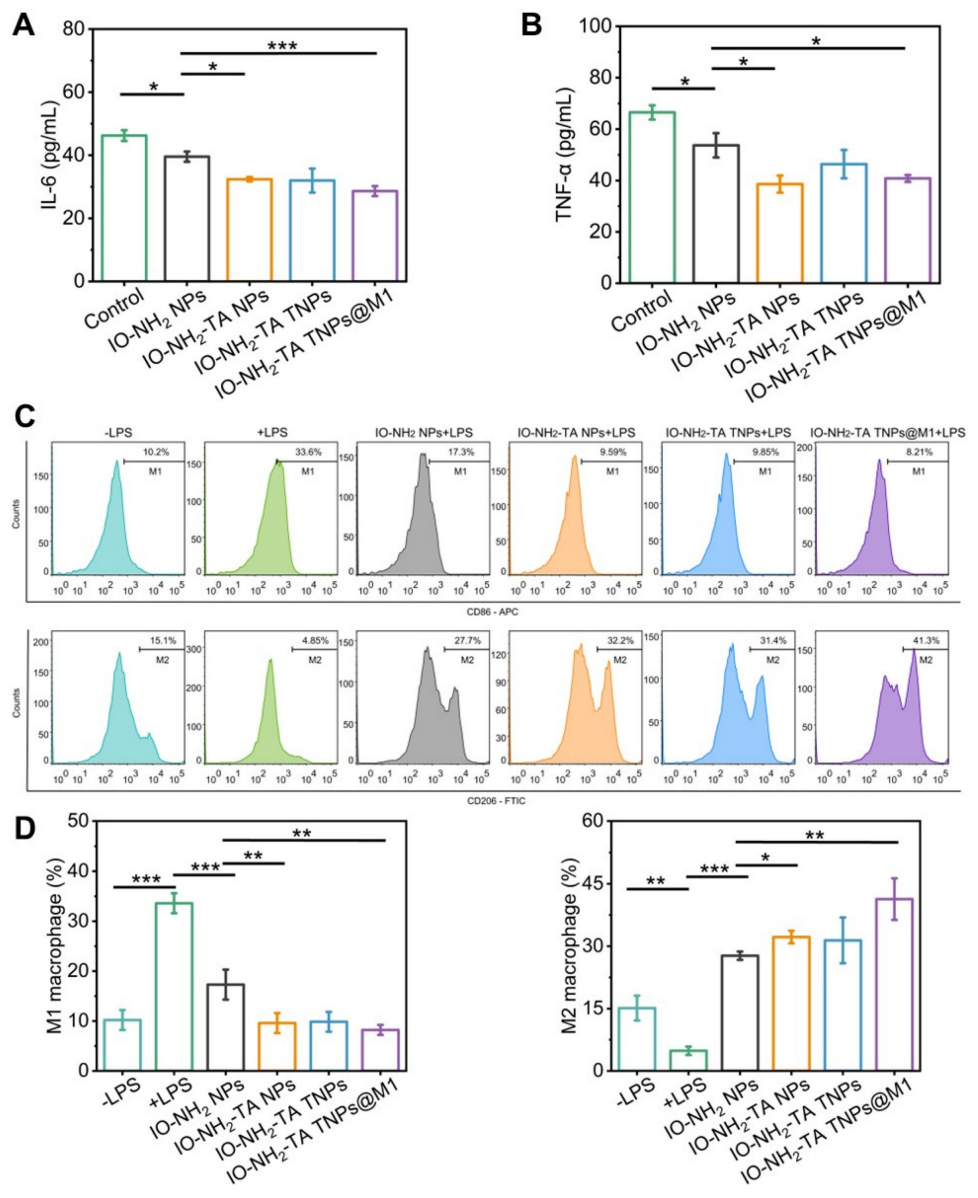
Expression levels of IL-6 (A) and TNF-α (B) in MH7H cells under various conditions. Flow cytometry analysis of CD86 and CD206 expression in macrophages (C) and quantitative analysis (D).

The anti-inflammatory efficacy of IO-NH_2_-TA TNPs@M1 was evaluated by measuring the levels of pro-inflammatory factors (IL-6 and TNF-α). Following treatment with IO-NH_2_-TA TNPs@M1, IL-6 content decreased from 46.25 ± 1.72 pg/mL to 28.64 ± 1.58 pg/mL (*p* < 0.05), and TNF-α content decreased from 63.72 ± 4.72 pg/mL to 40.83 ± 1.34 pg/mL (*p* < 0.05), indicating that IO-NH_2_-TA TNPs@M1 adsorbed pro-inflammatory factors, thereby improving the inflammatory microenvironment (Figure 4A, B). In addition to the enhanced Fenton reaction, this transformation was also attributed to Tof blocking of the signal transduction of inflammatory cytokines and M1 macrophage membranes adsorbed of pro-inflammatory factors at the lesion site 37,26.

LPS stimulation of RAW 246.7 cell was used to evaluate the effect of IO-NH_2_-TA TNPs@M1 nanoparticles on the macrophage polarization. M1 and M2 macrophages were labeled with CD86 and CD206, respectively. LPS stimulation increased the proportion of pro-inflammatory M1 macrophages and decreased the proportion of anti-inflammatory M2 macrophages (Figure 4C). In contrast, IO-NH_2_-TA TNPs@M1 decreased the proportion of M1 macrophages from 33.6% to 8.21%, while the proportion of M2 macrophages increased from 4.8% to 41.3% (*p* <0.01). The findings suggested that IO-NH_2_-TA TNPs@M1 successfully facilitated the transition of macrophages from the M1 to the M2 phenotype (Figure 4D). This transformation was primarily attributed to the ability of IO-NH_2_-TA TNPs@M1 to synergistically eliminate ROS via the Fenton reaction, resulting in the production of O_2_ 4.

### Characterization of IO-NH_2_-TA TNPs@M1DM

**Figure 5:**
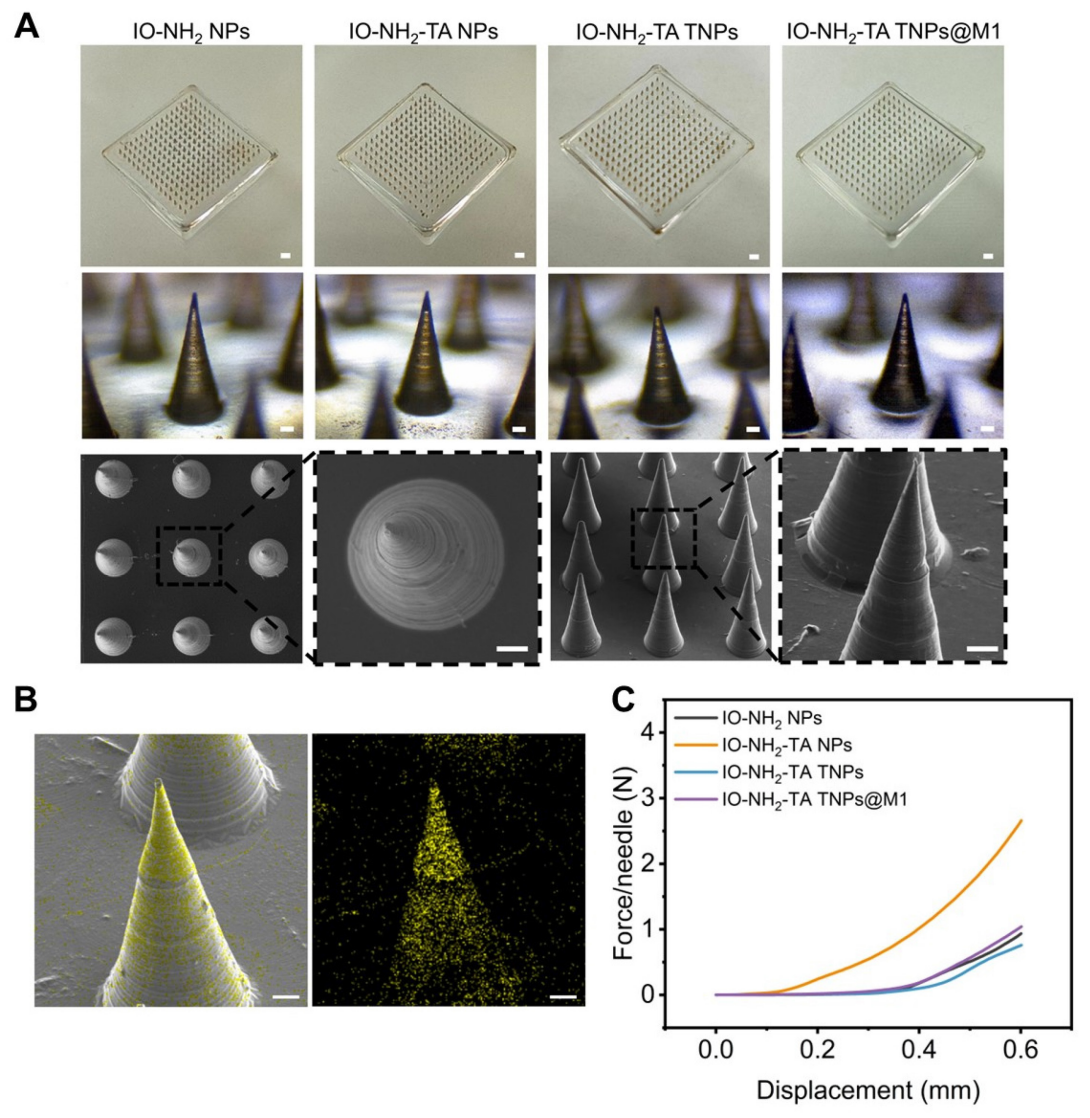
(A) Digital microscope images (scale bar, 1 mm), optical microscope images, and SEM images of IO-NH_2_-TA TNPs@M1DM (scale bar, 50 µm). (B) EDS mapping of IO-NH_2_-TA TNPs@M1DM. Scale bar, 50 µm. (C) Mechanical properties of microneedles.

The morphology of the microneedle was recorded by a digital camera, optical microscope and SEM. The microneedles were neat arranged in a 15×15 array, with a total of 225 needles (Figure 5A). The microneedles body was conical and black, about 600 µm high, and the base diameter was about 250 µm. EDS analysis indicated that iron oxide nanoparticles were successfully loaded and evenly distributed in IO-NH_2_-TA TNPs@M1DM to achieve continuous drug release (Figure 5B). Microneedles containing amphotericin B gels were also prepared using dissolvable polymers, which not only improved solubility of the drugs, but also overcame the problem of low bioavailability and side effects for oral administration 38.

In order to effectively penetrate the skin to achieve transdermal delivery, the microneedle had sufficient mechanical strength. In the deformation range of 0-0.6 mm, the curves presented a trend of slow growth at the beginning and rapid growth at the end, indicating that the change in microneedle shape increased with the increase in load (Figure 5C). Moreover, the curves were continuous, indicating that the microneedles were not broken and had excellent mechanical strength. When the shape variable reached a maximum, the required loads for the microneedle-loaded IO-NH_2_ NPs, IO-NH_2_-TA NPs, IO-NH_2_-TA TNPs and IO-NH_2_-TA TNPs@M1 were 0.94, 2.65, 0.76 and 1.04 N, respectively, which all were greater than the minimum force required to penetrate the skin (0.1 N). IO-NH_2_-TA NPs contained a large number of hydrophilic groups, softened after absorbing water, and possessed a decreased elastic modulus 39.

**Figure 6:**
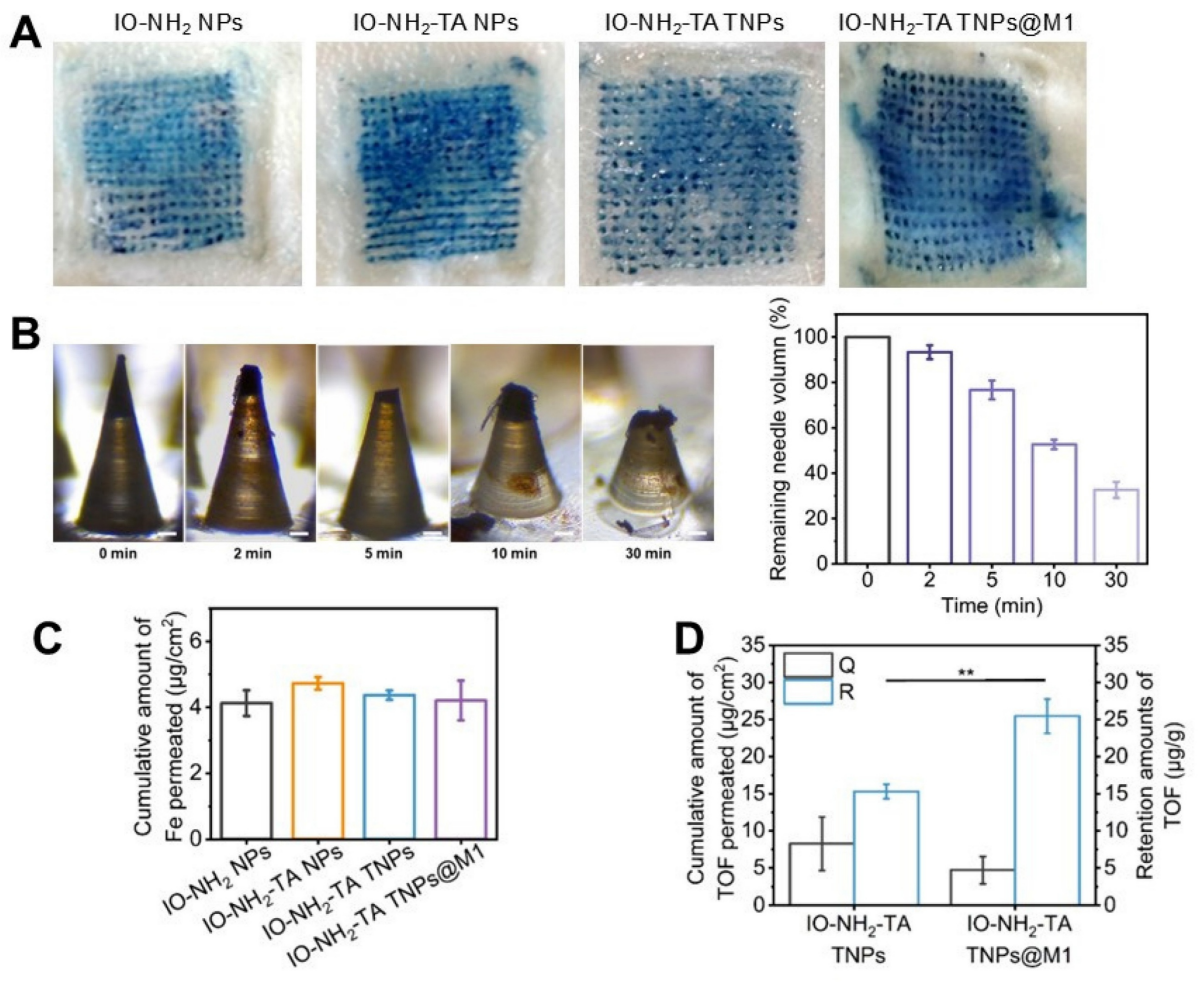
(A) Penetration of microneedles loaded with various microneedles (scale bar, 1 mm). (B) Mechanical properties of microneedles. Digital microscope images of IO-NH_2_-TA TNPs@M1DM at 0, 2, 5, 10 and 30 min after piercing into the rat skin *in vivo* (scale bar, 50 µm), and the percentage of undissolved volume of IO-NH_2_-TA TNPs@M1DM (*n* = 3). (C) Amounts of Fe in penetrated skin (*n* = 3). (D) Skin penetration and retention amounts of Tof (*n* = 3).

The ability of the microneedle to insert into skin was evaluated (Figure 6A). The microneedle containing methene blue were successfully inserted into rat skin, and produced the blue micropore array consistent with the number of microneedle bodies. The insertion efficiency of four microneedle was close to 100%. At 30 min, 68% of microneedle body were dissolved and released the nanoparticle (Figure 6B), indicating that the DM made of oligo-sodium hyaluronate had good mechanical properties for transdermal drug delivery, similar to the hydrophilic polysaccharides and hyaluronic acid 40.

Iron content is the key to inducing iron death in cells 41. The Fe content in microneedles loaded with IO-NH_2_ NPs, IO-NH_2_-TA NPs, IO-NH_2_-TA TNPs and IO-NH_2_-TA TNPs@M1 was 143.30 ± 13.03 μg, 145.38 ± 14.11 μg, 142.84 ± 10.30 μg and 144.55 ± 20.54 μg, respectively, which was close to the theoretical content (of 143 μg). The amounts of Fe penetrating the skin from different microneedles were basically same (*p* > 0.05, Figure 6C). The content of Tof in IO-NH_2_-TA TNPs and IO-NH_2_-TA TNPs@M1 microneedles was 40.04 ± 5.12 μg and 42.39 ± 5.25 μg, respectively. The amounts of Tof in penetrated skin following microneedle loading of IO-NH_2_-TA TNPs and IO-NH_2_-TA TNPs@M1 were 8.25 ± 3.62 μg/cm^2^ and 4.72 ± 1.82 μg/cm^2^, respectively (*p* > 0.05, Figure 6D). These results show that the difference in efficacy of different microneedles is irrelevant for the content of iron and drug.

### Pharmacodynamic study of IO-NH_2_-TA TNPs@M1DM

**Figure 7:**
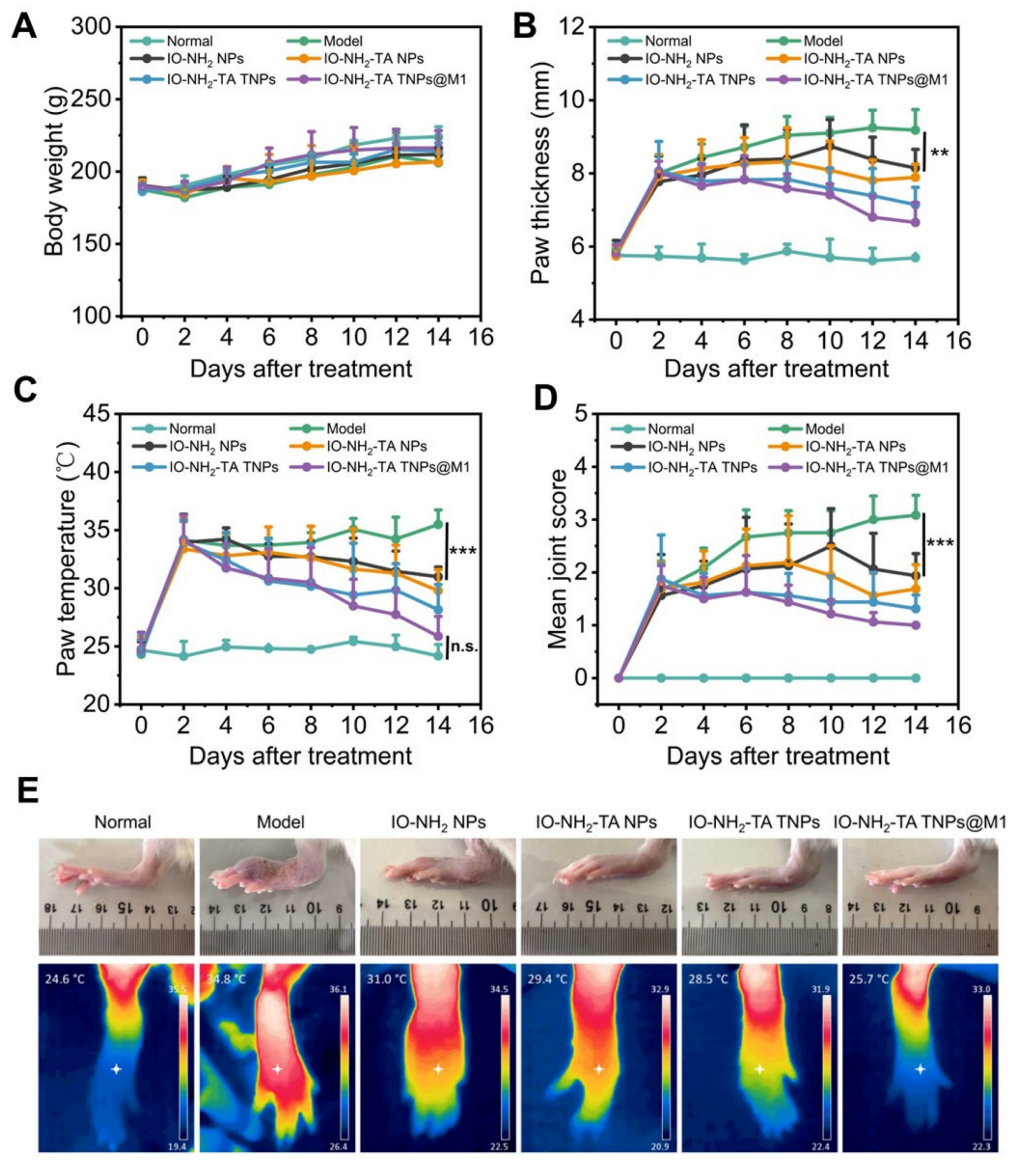
Body weight change (A), paw thickness (B), paw temperature (C) and arthritis joint scores of rats (D) under different treatments (*n* = 6). Rat joints at the end of different treatments (E).

During drug administration in animal models, the body weight of rats in each group gradually increased (Figure 7A), with no significant difference compared with the normal group (*p* > 0.05), indicating the relative safety of microneedles. Foot thickness and foot temperature of rats in the RA group increased (Figure 7B-D), as previously reported 42. All treatment groups had alleviated the progression of RA, with IO-NH_2_-TA TNPs@M1DM treatment having the best therapeutic effect, having foot thickness, foot temperature, and arthritis index reduced to 6.66 mm, 25.86 °C and 1.00, respectively (*p* < 0.01). Moreover, IO-NH_2_-TA TNPs@M1DM alleviated the swelling and joint fever to the greatest extent (Figure 7E).

### *In vivo* mechanisms of IO-NH_2_-TA TNPs@M1DM

**Figure 8:**
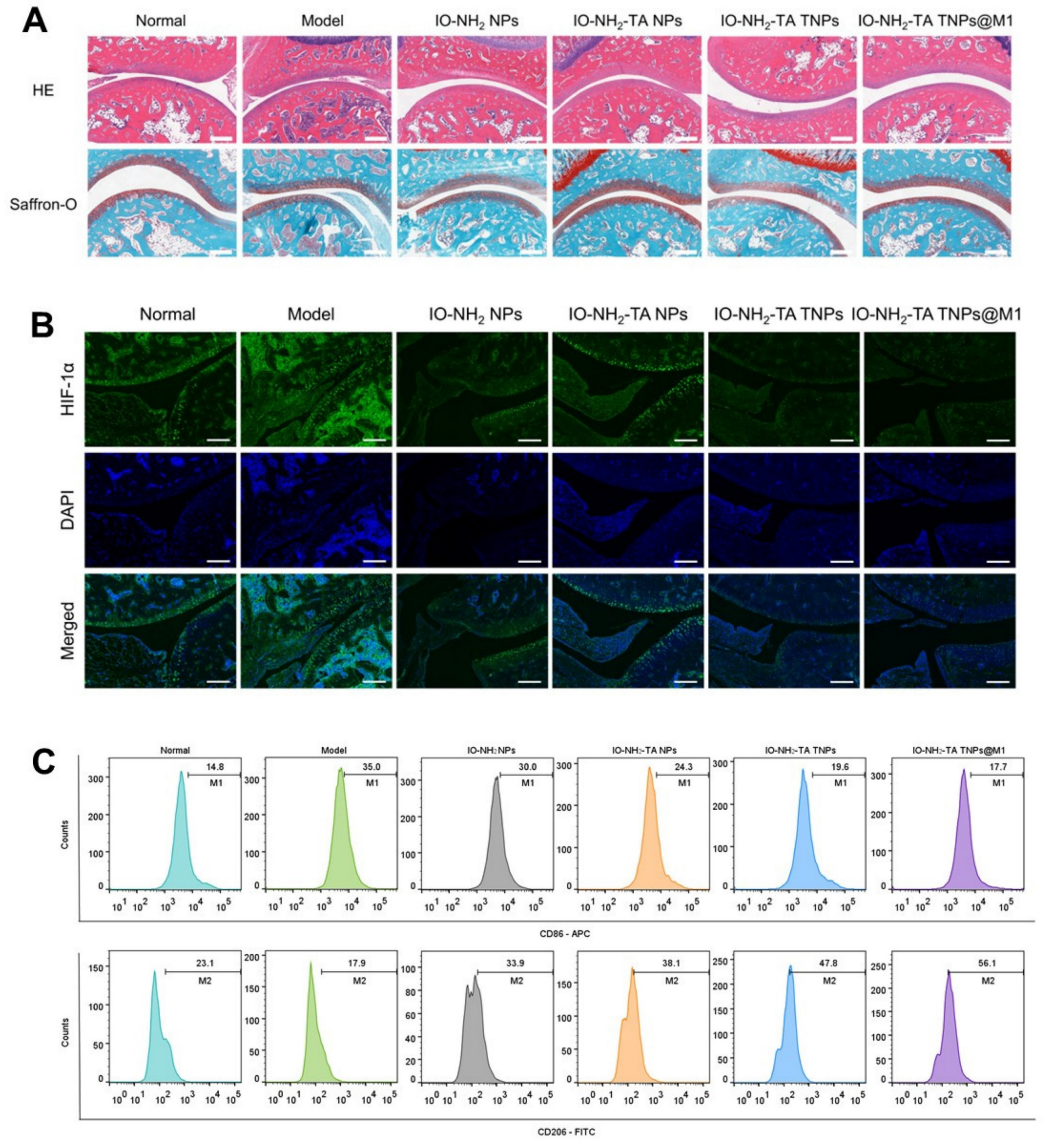
(A) HE staining, Safranin O solid green staining of rat joints following different treatments. Scale bar, 100 µm. (B) HIF-1α immunofluorescence staining of rat joints following different treatments. Scale bar, 200 µm. (C) Flow cytometry analysis of CD86 and CD206 expression in synovial tissue of rat joints.

H&E staining showed microneedle treatment relieved RA pathology (Figure 8A). The synovia of RA rats displayed inflammatory cell infiltration, hyperplasia and new capillary formation. After microneedle treatment, pathological symptoms were relieved to different degrees. IO-NH_2_-TA TNPs@M1DM treatment showed the most significant improvement on pathological symptoms of RA. To investigate changes in the extracellular cartilage matrix of articular cartilage, we used safranine O, which stains acidic glycosaminoglycans (orange-red), and solid green, which stains the nucleus and collagen fibers (blue-green) 43. In comparison to the normal group, the RA rats exhibited diminished safranine O staining, indicating a decreased distribution of proteoglycans and increased wear of the articular cartilage. Notably, the articular cartilage in the IO-NH_2_-TA TNPs@M1DM-treated rats closely resembled that of the normal group, displaying the most potent therapeutic effect compared to the other microneedles.

HIF-1α was a core driver of joint destruction by regulating the hypoxic microenvironment and inflammatory response. Immunofluorescence staining of ankle synovium of rats showed that, compared with normal group, HlF-1a expression level in the RA group was elevated, indicating that inflammation aggravated joint hypoxia (Figure 8B). IO-NH_2_-TA TNPs@M1DM treatment displayed the most pronounced inhibitory effect on HIF-1α expression, suggesting a profound reduction in joint hypoxia, similar to another study where AMSP-30m, another potential anti-arthritic compound, inhibited the expression of HIF-1α in CIA rat synovia and blocked activation of the sonic hedgehog pathway, thus revealing the pathogenic mechanism of the inhibition of fibroblast synovium cells 44.

Macrophage polarization plays a key role in the inflammatory response and joint destruction of RA 45. The proportion of M1 macrophages in RA rats increased and the proportion of M2 macrophages decreased, compared with normal group (Figure 8C). In contrast, IO-NH_2_-TA TNPs@M1 treatment decreased the proportion of M1 macrophages from 35.0% to 17.7%, while the proportion of M2 macrophages increased from 17.9% to 56.1%, indicating that IO-NH_2_-TA TNPs@M1 effectively induced the transformation of macrophages from M1 to M2. Another study used adipose stem cell exosomes loaded with icariin to promote M2 polarization by inhibiting the ERK/HIF-1α/GLUT1 pathway, which effectively alleviated cartilage destruction and synovial inflammation in RA rats 46.

### Safety evaluation of IO-NH_2_-TA TNPs@M1DM

**Figure 9:**
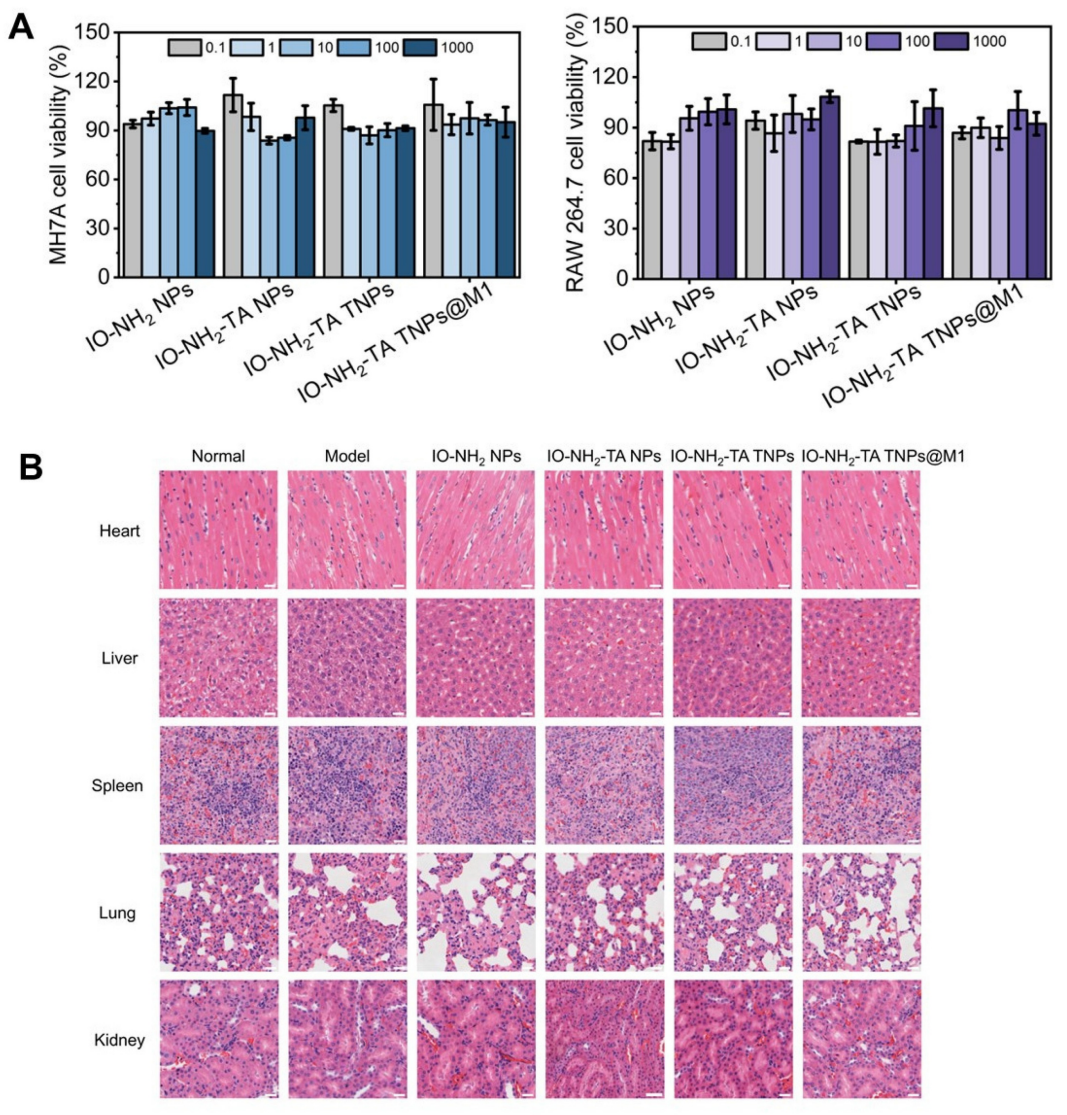
(A) Cytotoxicity of the different NPs on MH7A and RAW 264.7 cells. (B) Histopathological staining of major organs. Scale bar, 25 µm.

Firstly, cytotoxicity of the four types of nanoparticles was investigated (Figure 9A). In the concentration range of 0.1 to 1000 μg/mL, all four types of nanoparticles exhibited low toxicity (> 80%). Then, safety of the microneedles was evaluated by HE staining (Figure 9B). Compared with the normal group, the heart, liver, spleen, lung and kidneys of rats treated with different microneedles showed no significant pathological change. This finding further supports the conclusion that all microneedles demonstrate favorable biosafety profiles.

## Conclusion

In this study, a novel biomimetic self-enhancing Fenton reaction nano-reactor (IO-NH_2_-TA TNPs@M1) were designed and synthesized, which reduced ROS, inhibited HIF-1α expression, absorbed inflammatory factors and promoted M2 macrophage polarization. Then, the IO-NH_2_-TA TNPs@M1 was effectively incorporated into microneedles for transdermal delivery, resulting in a marked enhancement of the therapeutic efficacy for RA. From the "multi-target and multi-pathway", this study aimed to achieve a synergistic therapeutic effect by remodeling inflammatory microenvironment, which provided a new strategy for the clinical treatment of RA.

## Materials and Methods

### Chemicals and reagents

FeCl_3_·6H_2_O, FeSO_4_·7H_2_O, 3-aminopropyl triethoxysilane, TA, Tof, Triton X-100, DAPI, PVP-K90, and oligomeric sodium hyaluronate were purchased from Shanghai Macklin Biochemical Co., Ltd. (Shanghai, China). CD86-FITC antibody, APC-CD86, FTIC-CD206, and CD206-PE were purchased from ACROBiosystems Group (Beijing, China). DMEM, RPMI 1640, 2',7'-dichlorofluorescein diacetate, EDTA and fetal bovine serum were purchased from Dalian Meilun Biotechnology Co., Ltd. (Dalian, China). Anti-mouse FITC was from Shanghai Beyotime Biotechnology Co., Ltd. (Shanghai, China). Anti-β1 antibody and anti-β-actin antibody were purchased from Chengdu Zhengneng Biotechnology Co., Ltd. (Chendu, China). Goat anti-rabbit IgG H&L was from Wuhan Doctor De Biological Engineering Co., Ltd. (Wuhan, China). Complete Freund's adjuvant was from Sigma-Aldrich (USA). HIF-1α primary antibody was from Abbot (Shanghai) Trading Co., L td. (Shanghai, China). The PDMS mold was from Xiamen Qiepu Medical Technology Co., Ltd. (Xiamen, China).

### Synthesis and characterization of IO-NH_2_-TA TNPs

IO NPs were prepared by a coprecipitation method. FeCl_3_·6H_2_O and FeSO_4_·7H_2_O were dissolved in distilled water (0.5 mol/L). The above solutions (2:1) were mixed and stirred under the protection of nitrogen at 80 °C, then the pH of the reaction was adjusted to 11. The IO NPs were collected by magnetic separation and repeatedly cleaned with distilled water. Subsequently, the IO NPs were modified using a silane coupling agent (3-aminopropyl triethoxysilane) to obtain the IO-NH_2_ NPs. Then, IO-NH_2_ NPs were added into a TA solution, and magnetically stirred for 12 h, followed by removal of the free TA by dialysis to obtain IO-NH_2_-TA NPs. The IO-NH_2_-TA NPs were confirmed by ultraviolet spectrophotometry and transmission electron microscopy. IO-NH_2_-TA NPs were added to a Tof solution, magnetically stirred for 12 h, and the free Tof was removed by dialysis to obtain IO-NH_2_-TA TNPs. Drug loading and encapsulation efficiency of IO-NH_2_-TA TNPs were determined according to the ratio of drug to carrier mass of 1:1, 1:2, 1:5, 1:10 and 1:20, respectively.

### Synthesis and characterization of IO-NH_2_-TA TNPs@M1

The RAW264.7 cell line was procured from the Cell Bank of Chinese Academy of Sciences, and cultured in DMEM supplemented with 10% fetal bovine serum and 1% double antibody, and cultured at a temperature of 37 °C within an atmosphere containing 5% CO_2_. To induce macrophage polarization, lipopolysaccharide (LPS) was added for 24 h, which converted the macrophages to M1-type macrophages. The induced M1 macrophages were collected and suspended in TM buffer (pH 7.4, 10 mM Tris, 1 mM MgCl_2_) to a concentration of 2.5×10^7^ cells/mL. The cell suspension was repeatedly frozen and thawed three times, and cells were de-nucleated by an ultrasonic cell crusher for 5 min at 100 W power. Then, sucrose solution and cell homogenate were mixed, and centrifuged at 2000 g and 4 °C for 10 min. Supernatant was collected and further centrifuged for 30 min to obtain the cell membrane. The IO-NH_2_-TA TNPs solution (1 μg/mL) and M1 macrophage membrane (0.5 μg/mL) were mixed at 37 °C using an ultrasonic probe, and then the mixture was repeatedly squeezed by a membrane extruder to obtain the IO-NH_2_-TA TNPs@M1.

Western blotting was used to quantitatively characterize the expression level of specific proteins in IO-NH_2_-TA TNPs@M1, thus verifying whether M1 macrophage membrane was coated the nanoparticle. Particle size, distribution and zeta potential of the nanoparticles were measured with a Marvin laser particle size analyzer. The change in nanoparticle size in PBS and fetal bovine serum, with time, was investigated to examine the stability. The *in vitro* release curves of Tof from the nanoparticles were investigated by dynamic dialysis, in order to clarify whether drug release responded to RA microenvironment. PBS (pH 6.8) containing different concentrations of H_2_O_2_ (0, 0.01, 0.1 mM) were chosen.

### *In vitro* mechanisms of IO-NH_2_-TA TNPs@M1 on regulating the RA microenvironment

The RAW264.7 and MH7A cell lines were procured from the Cell Bank of Chinese Academy of Sciences, and were cultured in the DMEM (10% fetal bovine serum and 1% double antibody) and RPMI 1640 (10% fetal bovine serum and 1% double antibody), respectively. These cell lines were incubated at a temperature of 37 °C within an atmosphere containing 5% CO_2_.

2',7'-Dichlorofluorescein diacetate (DCFH-DA) was used as a fluorescent probe for detecting ROS, which was applied to investigate the scavenging ability of IO-NH_2_-TA TNPs@M1. MH7A cells were inoculated in a culture dish, then co-incubated with different nanoparticles (100 μg/mL) for 24 h. Finally, the above cells were stained with DCFH-DA (10 μM) for 20 min, and observed by confocal laser scanning microscope. The fluorescence intensity of the cells was measured by flow cytometry (Accuri™ C6).

Immunofluorescence staining for HIF-1α, was used to investigate the hypoxia-inhibitory ability of IO-NH_2_-TA TNPs@M1. MH7A cells were co-incubated with different nanoparticles (100 μg/mL) for 24 h. Culture medium was removed and fixed with 4% paraformaldehyde for 10min, permeabilized with Triton X-100 for 10 min, and blocked with 5% BSA at room temperature for 1 h. Then, HIF-1α primary antibody was incubated at 4 °C overnight, and FTIC-labeled secondary antibody was incubated at room temperature for 1 h. Cells were stained using DAPI and observed using confocal laser scanning microscopy.

The pro-inflammatory factor adsorption efficiency of IO-NH_2_-TA TNPs@M1 was studied by measuring IL-6 and TNF-α contents. The MH7A cells were incubated with different nanoparticles (100 μg/mL) for 24 h. Then the supernatants were collected and quantified by a Elx800 microplate reader.

The proportions of M2 macrophages were analyzed by detecting the surface marker (CD206), and was performed to investigate the polarization of M2 macrophages following treatment with IO-NH_2_-TA TNPs@M1. RAW 264.7 macrophages were treated with 1 μg/mL LPS for 24 h and then co-incubated with different nanoparticles (100 μg/mL) for 24 h. Cells were collected, labeled with FTIC-CD206 and APC-CD86 antibodies, and quantified by flow cytometry.

### Synthesis and characterization of IO-NH_2_-TA TNPs@M1DM

DMs were prepared using a molding method. Oligomeric sodium hyaluronates were added to a nanoparticle solution (50 mg/mL), then stirred and centrifuged at 4000 rpm for 5 min to remove bubbles, which was used as microneedle matrix solution. The matrix solution was added into a PDMS mold, centrifuged at 4000 rpm for 5 min to fill the pinholes, and dried in a dryer at room temperature for 2 h. Then backing solution (PVP-K90) was added and centrifuged at 4000 rpm for 5 min to form a backing layer. After the mold was dried in a dryer at room temperature for 24 h, the microneedles were removed from the mold.

The morphologies of different microneedles were recorded by a digital camera, optical microscope and scanning electron microscope (SEM), and EDS mapping of SEM was used to investigate the iron element surface distribution in the microneedles. The mechanical strength of the different microneedles was measured by an electronic universal testing machine (Instron 5969). The skin insertion ability of microneedles containing methylene blue was evaluated using rats, and observed under a microscope. The contents of Tof and iron were determined by UPLC and ICP/MS, respectively. *In vitro* drug skin penetration behavior in different microneedles was investigated using a vertical Franz diffusion cell.

### Pharmacodynamic study of IO-NH_2_-TA TNPs@M1DM

Wistar rats (200 ± 20 g) were provided by the Laboratory Animal Center of Shantou University Medical College (Guangdong, China). Adjuvant-induced RA models were developed with complete Freund's adjuvant (10 mg/mL) 47. Except for the control group, other rats were subcutaneously injected in left and right side with 0.05 mL CFA. Afterward, the rats were placed at constant temperature in a clean environment, and provided with sufficient food and water. Significant redness and swelling of the feet were regarded as the signs of successful RA induction. RA rats were randomly divided into 5 groups (*n* = 6): RA model group, IO-NH_2_ NPs DM group, IO-NH_2_-TA NPs DM group, IO-NH_2_-TA TNPs DM group and IO-NH_2_-TA TNPs@M1DM group. Six rats were selected as the normal group. For microneedle administration, microneedles were pressed for 1 min on the hairless leg skin of rats, and then fixed with a medical tape. Drugs were administered once every two days for 7 consecutive times.

The weight and foot thickness were measured, and foot temperature was monitored with near-infrared thermal imaging. The arthritis index was scored using a 5-level scoring method, including 0 (normal joints or without swelling), 1 (mild redness or swelling of plantar joint), 2 (moderate swelling of plantar or ankle), 3 (severe swelling of the ankle joint or total swelling below the ankle joint) and 4 (swelling of the whole paw or severe deformation of the joint).

### *In vivo* mechanisms of IO-NH_2_-TA TNPs@M1DM

Hematoxylin-eosin (HE) staining was used to observe the histopathological changes of synovitis, which reflected the intervention of microneedles on synovitis. After pharmacodynamic experiments, the rats were killed and the posterior ankle joints were removed, then washed with normal saline and fixed with 4% paraformaldehyde for 24 h. Subsequently, decalcification was performed with 10% EDTA. After decalcification, tissues were rinsed with distilled water for 30 min, then stained and observed under a microscope.

Joint sections were stained for HIF-1α immunofluorescence to investigate the effects of different microneedles on degree of hypoxia. Other experimental procedures were in accordance with Section: *In vitro* mechanisms of IO-NH_2_-TA TNPs@M1 on regulating the RA microenvironment.

The effects of different microneedles on macrophage polarization were investigated by CD86-FITC and CD206-PE staining. After treatment, synovia of rat joints were harvested and minced, then digested with collagenase Ⅳ. The cells were collected, and incubated with FITC-conjugated anti-CD86 (1:100) and PE-conjugated anti-CD206 (1:100) antibodies. Finally, the cells were examined using flow cytometry to calculate the percentage of CD86^+^ or CD206^+^ cells.

### Safety evaluation of IO-NH_2_-TA TNPs@M1DM

The cytotoxicity of different nanoparticles was quantified using MH7A and RAW 264.7 cells. Because microneedles cause cytotoxicity or mechanical damage, microneedle cytotoxicity experiments were not performed. Cells were inoculated in 96-well plates and co-incubated with nanoparticle-containing solution at different concentrations (0.1~1000 μg/mL) for 24 h. Then, CCK-8 reagent was added and cells were incubated for 30 min, then viability was measured with a microplate reader.

HE staining was used to evaluate the biosafety of different microneedles. After the experiments, the rats were killed, and the hearts, livers, spleens, lungs and kidneys were taken, fixed with 4% paraformaldehyde, embedded in paraffin wax and stained with HE, and the changes of histopathological structure were observed under a microscope.

### Statistical analysis

Quantitative results are statistically expressed as the mean ± standard deviation. Statistical analyses, including Student's t-test and one-way analysis of variance (ANOVA), were performed using GraphPad Prism 8.0 software (GraphPad, Inc., La Jolla, CA, USA). Statistical thresholds for significance were established as *p* < 0.05, with levels denoted as *p* < 0.05 (*), *p* < 0.01 (**) and *p* < 0.001 (***), showing varying degrees of confidence.

## Supplementary Material

Supplementary figures and table.
