# Supplementary material for: Transdermal microneedle integrating a biomimetic self-enhancing Fenton reaction nano-reactor for alleviating rheumatoid arthritis by inflammatory microenvironment remodeling
Source: Theranostics. 2025 Jun 18;15(14):7180–96. doi: 10.7150/thno.114855 (PMC12204083; doi:10.7150/thno.114855)
Supplement: Supplementary file 1 — Supplementary figures and table. [file thnov15p7180s1.pdf]

Supporting Information *for*

Transdermal microneedle integrating a biomimetic self-enhancing Fenton reaction nano-reactor for alleviating rheumatoid arthritis by inflammatory microenvironment remodeling

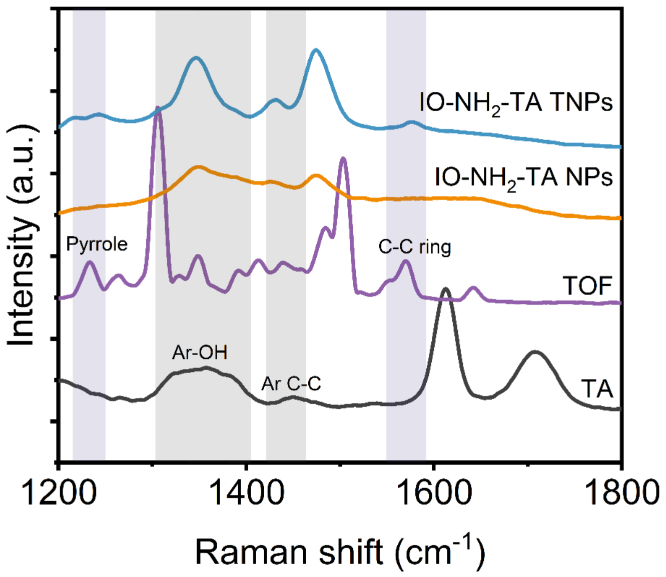

Figure S1. The Raman spectra of TA, TOF, IO-NH2 NPs and IO-NH2-TA TNPs.

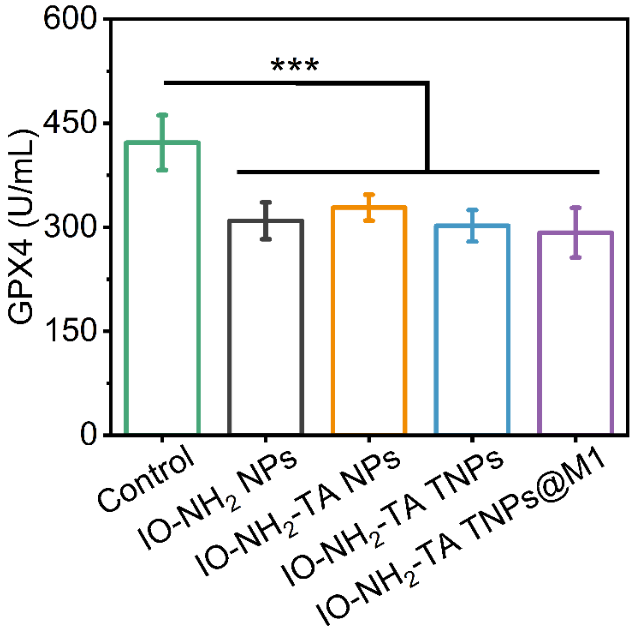

Figure S2. Expression levels of GPX4 in MH7H cells under various conditions.

Table S1 The particle size and zeta potential of $IO\text{-}NH_2$ NP、$IO\text{-}NH_2$-TA NPs、$IO\text{-}NH_2$-TA TNPs and $IO\text{-}NH_2$-TA TNPs

Name	Particle size (nm)	*PDI*	*zeta* potential (mV)
$IO\text{-}NH_2$ NPs	218.4 ± 4.2	0.096 ± 0.012	0.00 ± 0.12
$IO\text{-}NH_2$-TA NPs	235.4 ± 6.3	0.154 ± 0.033	-37.97 ± 2.34
$TO\text{-}NH_2$-TA TNPs	276.5 ± 8.8	0.148 ± 0.046	-28.81 ± 3.01
$IO\text{-}NH_2$-TA TNPs@M1	294.5 ± 11.2	0.096 ± 0.039	-32.34 ± 1.97
